# 

**Published:** 1885-09

**Authors:** 


					﻿io cts. a copy.
SEPT., 1885.
$i oo per year.
gJOWAL'J
HEALTH
ESTABLISHED 1854
Vi'3 2
c^e.- 9
OFFICE, 75 & 77 BARCLAY STREET, NEW YORK.
Entered as Second-class Matter at the Post-Office, New York.
				

